# Notes and News

**Published:** 1899-04-08

**Authors:** 


### Notes and News.

(Collected and Communicated.)

### HOSPITAL MEETINGS.

**Miller Hospital and Royal Kent Dispensary, Greenwich.—Annual General Meeting, at eight p.m., on Monday, April 10th, to be held in the Board Room of the Dispensary, Greenwich Road.**

An outbreak of cerebro-spinal meningitis has recently occurred in Philadelphia, 34 cases with 13 deaths having been reported in one week. The South-western States have also suffered much from the same disease during the past winter. Upwards of sixty deaths from this cause were reported in St. Louis during the month of February.

When addressing a meeting recently, Mr. John Burns, M.P., made the suggestion that, in order that large companies employing labour should not escape the responsibility of contributing to the hospitals, a stamp duty should be imposed upon shareholders. Mr. Burns' statement that the contribution to the metropolitan hospitals through the Hospital Sunday Fund does not exceed twopence per head of the population is incontestable; but it is to be hoped that each individual will realise his responsibility and subscribe voluntarily rather than that any arbitrary levy should be made, which would destroy sympathy and interest, and prove the thin edge of the wedge of State control.

The people of the United States are waking up to the fact that rabies is an actual disease and that there really is such a malady as hydrophobia. The New York *Medical News* says that for various reasons a certain amount of doubt has existed in America, even among physicians, as to the occurrence of genuine cases of hydrophobia in that country, and describes the amusement which was caused among the Russian physicians at the International Medical Congress in Moscow when an American medical visitor said that there were doctors in America who doubted the existence of hydrophobia. However, Dr. Cabot, assistant bacteriologist to the city of New York, has made it pretty plain that not only rabies but hydrophobia are realities in that State. Unfortunately his experience has also made it evident that there are people in the United States quite as impervious to reason on the question of the scientific investigation of disease as even the most fervent opponents of science in this country.

At an inquest held at the Bermondsey Town Hall on Saturday on a man, aged 26, some very strong remarks were made by the deputy coroner about the difficulties which are made by the workhouse authorities about taking into the Poor-law infirmaries patients sent on from the hospitals. "It is utterly absurd and cruel to poor people," he said, "that they cannot be admitted direct to the workhouse or infirmary on the recommendation of the hospital authorities without being carted about to relieving officers. It is a scandalous thing, and ought to be put a stop to." It appeared that on the patient being taken to Guy's Hospital the case was diagnosed as acute melancholia after influenza, and as cases of mental disease were not allowed to be admitted into the hospital the case was sent on to the infirmary. In the end the master of the workhouse, although still thinking that the hospital should detain such cases and not send them to the infirmary, undertook to admit them on his own responsibility, and without a relieving order, if they were sent on with a recommendation from the hospital. This seems a common-sense arrangement.

The Rochford Guardians have got themselves into a difficulty. Improvements and additions to their infirmary became necessary, and apparently a tender to execute the work was accepted without very much knowledge having been brought to bear on the subject. A recent meeting of the board revealed great diversity of opinion on the subject of expenditure, one gentleman estimating £1,000 to be sufficient, whilst a tender for £7,000 has been accepted. One guardian admitted "they were in a hole," and the decision arrived at was an adjournment of the discussion. Those concerned in establishing and maintaining institutions for the sick are gradually becoming alive to the fact that expert knowledge saves expense in the end, but there are still too many who are ready to take up duties which they do not trouble to understand. There is much literature published nowadays to shed light upon expenditure, and comparisons are not difficult to make. At Rochford they require nurses' quarters, new wards, and improved drainage. It is to be hoped someone will be consulted who is capable of directing the guardians as to what they really should spend.

At the annual conference of the Friendly Societies' Medical Alliance, which was held at Leamington on April 3rd, the President, while expressing regret at the hostility of members of the medical profession to their aims and objects, had to admit that, if the General Medical Council had taken the extreme step of striking off the register the names of all medical men serving such associations as guilty of "unprofessional conduct," their association would have been placed in a very difficult position, even if it had continued to exist at all. This, however, had not been done, and a proposal had been made to form a reconciliation board for the settlement of differences between friendly society medical associations and their medical officers. It is, however, to be noted that although he held that the associations would have to reserve their membership exclusively to members of registered friendly societies and their families, he also held absolutely to the right of the friendly societies to make combined provision for medical attendance for all their members altogether apart from their social position. As members of friendly societies, they would strenuously resist any claim put forward to impose any wage or other limit to membership of such societies. This being so, we may presume that all the talk about "reconciliation boards" is but blarney.

